# Investigation of the Resistivity and Emissivity of a Pellicle Membrane for EUV Lithography

**DOI:** 10.3390/membranes12040367

**Published:** 2022-03-26

**Authors:** Seong Ju Wi, Yong Ju Jang, Haneul Kim, Kyeongjae Cho, Jinho Ahn

**Affiliations:** 1 Division of Materials Science and Engineering, Hanyang University, Seoul 04763, Korea; wsj1992@hanyang.ac.kr (S.J.W.); skylife103@hanyang.ac.kr (H.K.); 2 EUV-IUCC (Industry University Cooperation Center), Hanuyang University, Seoul 04763, Korea; abco2560@gmail.com; 3 Division of Nanoscale Semiconductor Engineering, Hanyang University, Seoul 04763, Korea; 4 Department of Materials Science and Engineering, University of Texas at Dallas, Richardson, TX 75080, USA; kjcho@utdallas.edu

**Keywords:** EUV pellicle, emissivity, Lorentz–Drude model, resistivity, grain size, membrane

## Abstract

A pellicle is a thin membrane structure that protects an extreme ultraviolet (EUV) mask from contamination during the exposure process. However, its limited transmittance induces unwanted heating owing to the absorption of EUV photons. The rupture of the EUV pellicle can be avoided by improving its thermal stability, which is achieved by improving the emissivity of the film. However, the emissivity data for thin films are not easily available in the literature, and its value is very sensitive to thickness. Therefore, we investigated the dependence of emissivity on structural parameters, such as thickness, surface roughness, and grain size. We found a correlation between resistivity and emissivity using theoretical and experimental approaches. By changing the grain size of the Ru thin film, the relationship between resistivity and emissivity was experimentally verified and confirmed using the Lorentz–Drude model. Finally, we present a method to develop an EUV pellicle with better thermal stability that can withstand high-power EUV light sources.

## 1. Introduction

Extreme ultraviolet (EUV) lithography has been applied to the high-volume manufacturing (HVM) of semiconductor logic devices and dynamic random-access memory (DRAM) at 7-nm technology nodes and beyond [1,2]. The EUV pellicle is a free-standing membrane that protects the EUV mask from the external defects generated during the exposure process, thus improving the yield of the EUV lithography process [3,4]. The EUV pellicle requires a transmittance higher than 90% at a 13.5-nm wavelength to minimize the loss of throughput caused by the absorption of EUV photons by the pellicle. In addition, the mechanical, chemical, and thermal durability of the pellicle is essential inside an EUV scanner [5,6,7,8,9]. In particular, excess heating due to the absorption of EUV photons can destroy the pellicle membrane due to thermal stress, which is fatal to the availability of an EUV scanner [10]. Therefore, it is necessary to keep the EUV pellicle intact by improving the cooling efficiency of the pellicle material.

Generally, a material can be cooled by conduction, convection, or radiation [11]. However, the cooling efficiencies by conduction and convection are very limited because of the thin membrane structure of the EUV pellicle and the high-vacuum environment of the EUV scanner, respectively. Therefore, the EUV pellicle is mainly cooled by radiation [12], and ensuring the thermal emissivity of the pellicle structure is important [13,14,15]. However, the emissivity values of nanoscale thin films are not easily available, and the measurement of emissivity is not intuitive.

Herein, the improvement of cooling efficiency is demonstrated by changing the grain size of the ruthenium thin film. In addition, the relationship between the resistivity and emissivity of the Ru thin film is experimentally verified and theoretically confirmed based on the Lorentz–Drude model. Finally, it is argued that emissivity can be improved by changing the microstructure in the direction of increasing the resistivity of the thin film.

## 2. Theoretical Approach

### 2.1. Emissivity and the Lorentz–Drude Model

According to Kirchhoff′s law, emissivity is equal to absorbance (α), which can be calculated from reflectance (R) and transmittance (τ) using the energy conservation law, as shown in Equation (1) [16].

(1) α(λ,T)=1−R(λ,T)−τ(λ,T).

As temperature increases, the total radiated energy of a body increases and the peak of the emitted spectrum shifts to a shorter wavelength according to Planck′s law, which describes black body radiation [17]. During the exposure process, the EUV pellicle is heated to hundreds of degrees Celsius such that the emitted spectrum is mainly generated in the infrared (IR) wavelength region. Therefore, the emissivity of the EUV pellicle can be calculated from the average absorbance of the IR wavelength region [13].

Furthermore, transmittance and reflectance are related to the refractive index and the extinction coefficient, which can be calculated using complex permittivity (*ε*) [18,19,20]. Complex permittivity is derived from the Lorentz–Drude model, as shown in Equation (2).

(2) $$\varepsilon \left(\omega \right)=1+\varepsilon_{Drude}+\underset{n}{\sum }\varepsilon_{Lorentz}.$$

The overall optical response follows the Lorentz oscillator model because the inter-band transition is dominant in the ultraviolet and visible regions. Meanwhile, the Drude model mainly determines the optical constants in the IR wavelength region because the intra-band transition is dominant owing to the free electron contribution [21,22].

Because the thermal emission layer of the EUV pellicle is generally conductive and the emitted spectrum of the EUV pellicle is primarily in the IR region during the exposure process, the emissivity of the EUV pellicle is determined by the Drude model.

(3) $$\varepsilon_{Drude}\left(\omega \right)=\varepsilon_{\infty }-\frac{\omega_{p}^{2}}{-\omega^{2}-i\frac{\omega }{\tau }}$$

(4) $$\tau =\frac{m_{0}}{\rho ne^{2}}$$

Therefore, complex permittivity is related to plasma frequency ($\omega_{p}$) and electron scattering time (τ) using the Drude model, as shown in Equation (3). Plasma frequency is unique for each material, and electron scattering time can be derived as shown in Equation (4). Here, ρ is the resistivity of the film, e is the electron charge, n is the electron concentration, and $m_{0}$ is the electron mass. From this equation, the inverse relationship between electron scattering time and resistivity can be confirmed. In addition, the change in resistivity is expected to directly affect electron scattering time and subsequently emissivity [22,23].

### 2.2. Thin Film Resistivity Model


(5) $$\rho =\rho_{i}\left[1+\frac{0.375\left(1-p\right)Sl}{d}+\frac{1.5Rl}{\left(1-R\right)g}\right]$$

Equation (5) represents a resistivity model for thin films that considers the bulk resistivity and the effect of surface and grain boundary scattering [24,25,26]. Here, d is the film thickness, l is the mean free path of an electron, and g is the grain size. The factor S corresponds to the surface roughness factor, which is equal to 1 for a perfectly flat film surface and increases as the surface becomes rougher. Moreover, p is the specularity parameter, which is related to the surface roughness factor, and R is the scattering coefficient, which indicates the probability that an electron is scattered at a grain boundary. Therefore, variables such as the surface roughness, grain size, and film thickness are the main factors that influence the resistivity, which subsequently affect the emissivity of the EUV pellicle.

## 3. Experimental Methods

### 3.1. Sample Preparation and Analysis

A full-size (110 × 144 mm) EUV pellicle is required to sufficiently protect the EUV mask from external defects during the EUV exposure process. However, it is technically challenging to fabricate a full-size pellicle membrane as the pellicle is an ultra-thin film with a thickness of only a few tens of nanometers. Therefore, the measurement and evaluation were performed on a small-sized (10 × 10 mm) pellicle. However, it is expected that a similar dependency will be observed even with a full-size pellicle.

**Figure 1:**
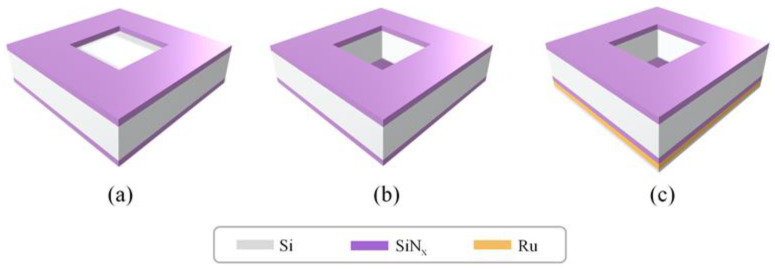
Fabrication of the Ru/SiN_x_ pellicle composite: (**a**) SiN_x_ film deposition using low-pressure chemical vapor deposition (LPCVD) and backside patterning by reactive ion etching, (**b**) fabrication of the SiN_x_ free-standing membrane using the Si wet etching process, (**c**) Ru layer deposition by magnetron sputtering and annealing at 300 and 500 °C using a vacuum furnace.

Figure 1 presents a schematic of the fabrication process for the Ru/SiN_x_ pellicle composite used in this study. A 40-nm-thick silicon nitride (SiN_x_) film was deposited by low-pressure chemical vapor deposition (LPCVD) onto a 725-μm-thick (100) silicon wafer using ammonia (NH_3_) and dichlorosilane (DCS, SiH_2_Cl_2_) gas at 830 °C. Subsequently, a DPD-200 photoresist was coated on the backside, and photolithography was performed. This was followed by reactive ion etching using CF_4_, CHF_3_, and O_2_ gas to form a backside window for wet etching. Furthermore, free-standing SiN_x_ membranes with a size of 10 × 10 mm were fabricated by silicon wafer back-etching in a 30 wt% potassium hydroxide (KOH) solution at 60 °C. A 4-nm-thick Ru film was then deposited onto the SiN_x_ free-standing membrane by DC magnetron sputtering. The target used was a 4-inch disk of Ru (99.95%) metal, and the chamber was evacuated to a base pressure of less than 7 × 10^–7^ Torr prior to deposition. The Ru films were deposited in pure Ar gas (99.9999%) at a pressure of 10^–3^ Torr, and the substrate temperature was kept constant at 25 °C. Thereafter, the Ru/SiN_x_ pellicle composite was annealed at 300 and 500 °C for 30 min in a gas mixture of 96% Ar and 4% hydrogen (H_2_) of 99.999% purity in a vacuum furnace. The chamber was evacuated to a base pressure of 5 × 10^–3^ Torr, and the flow rate of the Ar and H_2_ mixture was fixed at 1 sccm. The heating rate was fixed at 10 °C min^–1^, and cooling was also done in a vacuum environment.

The thickness of the Ru film with respect to the annealing temperature was confirmed by a cross-view image acquired using transmission electron microscopy (TEM, JEM 2100F, JEOL, Tokyo, Japan). To compare the grain size, top-view TEM images of the Ru films deposited on SiN_x_ grids (21515-10, TED PELLA, Redding, CA, USA) were obtained, and the sizes of 40 grains were averaged. Atomic force microscopy (AFM, XE-100, Park systems, Suwon, Korea) was used to confirm the surface roughness for a 1 × 1 μm probing area. Lastly, the sheet resistance of the Ru film was measured with a 4-point probe and multiplied with the film thickness to confirm the resistivity.

### 3.2. Heat Load Test

**Figure 2:**
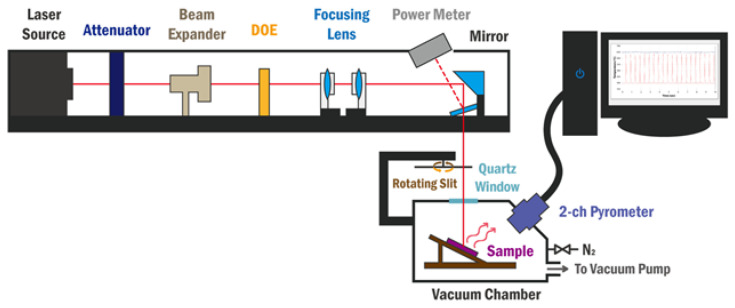
Schematic of the heat load test equipment.

Figure 2 shows a schematic of the heat load test equipment used to evaluate the thermal properties of the EUV pellicle composite. The thermal properties were evaluated by measuring the membrane temperature according to the absorbed heat load when a 355-nm ultraviolet (UV) laser was exposed to the membrane.

**Figure 3:**
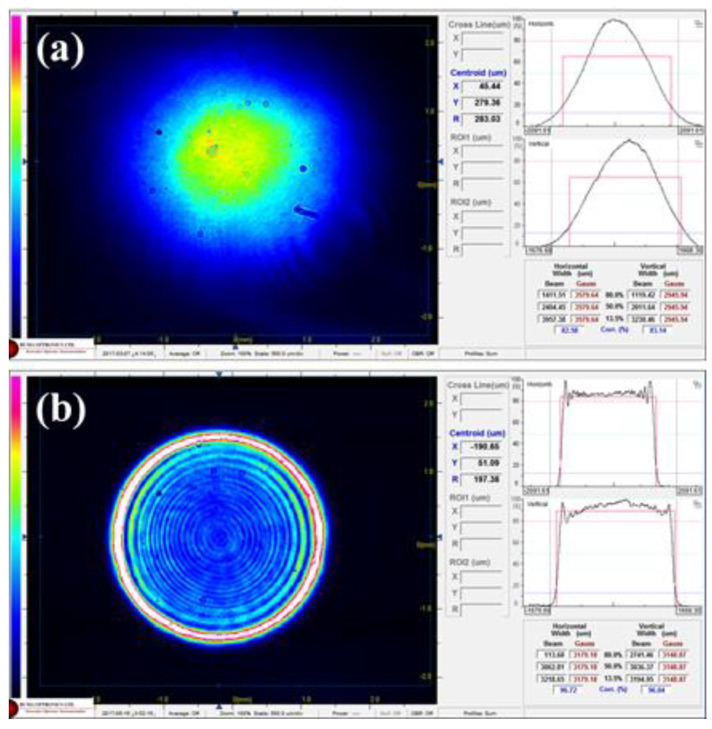
(**a**) Gaussian and (**b**) top-hat profile of the 355 nm UV laser measured by a beam profiler.

During the exposure, EUV light was incident on the pellicle with uniform intensity. An environment similar to the exposure process is required to obtain the reliability of the heat load test. Therefore, the Gaussian beam profile of the UV laser was tuned to a top-hat profile with uniform intensity by applying a diffractive optical element, as shown in Figure 3. To emulate the EUV exposure, the chamber was evacuated to less than 6 × 10^–6^ Torr, and EUV pellicle was exposed to 0.1-s heating and 0.9-s non-heating conditions per cycle by the rotating slit at the top of the vacuum chamber. In this study, the heat load test was performed for 60 cycles at various absorbed heat loads.

(6) $$I_{abs}=\frac{P}{D}\times \alpha$$

The absorbed heat load of the pellicle composite by the UV laser was calculated using Equation (6). Here, P is the laser power, D is the area of the incident laser, and α is the absorbance of the pellicle composite at a wavelength of 355 nm. In this study, D was kept constant at 6 mm. Thereafter, the absorbed heat load was calculated by measuring the absorbance of the pellicle composite using a UV-visible spectrophotometer. The temperature of the pellicle composite was measured using a 2-channel pyrometer during the heat load test, and the average of the peak temperature was calculated. The measurement accuracy of the 2-channel pyrometer was ±2%, and the detection range was 400–1500 °C.

Furthermore, the heat load test was performed in a high-vacuum environment, and the pellicle composite was at the nanometer scale thickness. Therefore, the cooling mechanism by convection and conduction can be excluded and the emissivity can be evaluated from the heat load test results. The emissivity of the pellicle composite was calculated from the absorbed heat load and average peak temperature using the heat transfer equation shown in Equation (7), wherein the effects of heat conduction and convection were excluded [12,24,25].

(7) $$\frac{dT}{dt}=\frac{1}{c\cdot m}\cdot \left[\alpha \cdot P-\epsilon \cdot \sigma \cdot S\cdot \left(T^{4}-T_{s}^{4}\right)\right]$$

Here, c is the specific heat, m is the mass of membrane, ϵ is emissivity, σ is Stefan–Boltzmann constant (5.67 × 10^–8^ Wm^−2^ K^−1^), T is the temperature of membrane, and $T_{s}$ is the temperature of the surrounding air.

## 4. Results and Discussion

### 4.1. Resistivity Parameter Analysis

**Figure 4:**
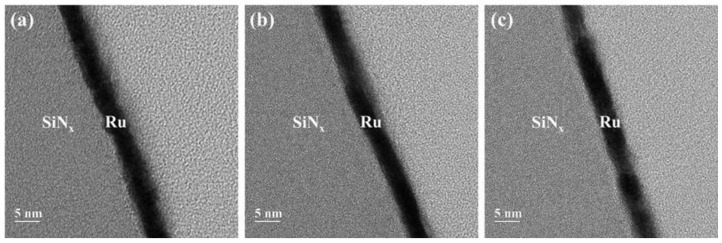
Cross-sectional TEM images of the Ru/SiN_x_ pellicle composites: (**a**) as deposited, (**b**) after annealing at 300 °C, and (**c**) after annealing at 500 °C.

**Figure 5:**
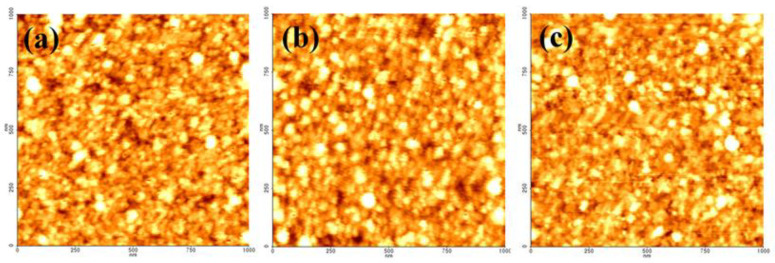
AFM images of the Ru films: (**a**) as deposited, (**b**) after annealing at 300 °C, and (**c**) after annealing at 500 °C.

Figure 4 shows the cross-sectional TEM images of the Ru/SiN_x_ composite membranes as deposited and after annealing at 300 and 500 °C. Under each annealing condition, the thickness of the Ru thin film was maintained at approximately 4.1 nm. Figure 5 presents the surface roughness of the Ru film as a function of annealing temperature, and the root-mean-square values of the surface roughness were 0.775, 0.805, and 0.709 nm, respectively. The thickness and surface roughness are factors that affect the surface scattering of the thin metal film according to Equation (5) [26,27]. In this study, the effect of surface scattering on the change in resistivity was excluded because the corresponding factor was constant at varying annealing temperatures.

**Figure 6:**
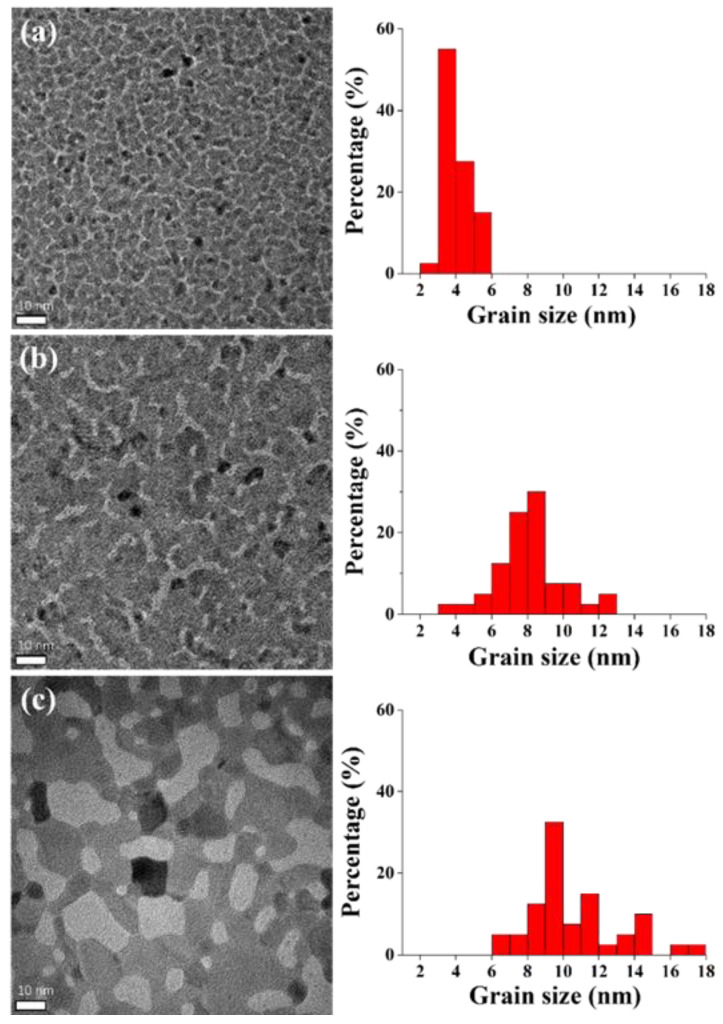
TEM top-view images and the average grain sizes of the Ru films for 40 grains: (**a**) as deposited, (**b**) after annealing at 300 °C, and (**c**) after annealing at 500 °C.

Figure 6 shows the calculated average grain size of the Ru films after annealing treatment. The average grain size increased from 4.0 to 8.1 and 10.6 nm owing to the grain growth due to annealing at 300 and 500 °C, respectively. According to Equation (5), the grain boundary scattering is inversely proportional to the grain size [26,27]. Therefore, the resistivity of the Ru film was expected to decrease as the annealing temperature increased.

### 4.2. Resistivity and Emissivity

**Figure 7:**
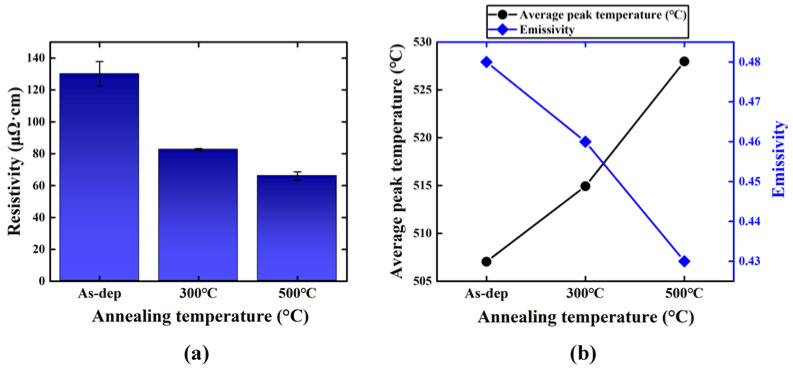
(**a**) Resistivity, and (**b**) peak temperature measured from the heat load test and calculated emissivity of the Ru/SiN_x_ pellicle composite: As deposited, after 300 °C annealing, and after 500 °C annealing.

Figure 7a presents the results of the resistivity measurements for the Ru thin films. Due to the annealing treatment, the resistivity decreased from 130 to 83 and 66 μΩ·cm, which was expected from the grain boundary scattering model. Figure 7b shows the heat load test results for the Ru/SiN_x_ pellicle composites under the same absorbed heat load of 1 W cm^–2^. After the annealing process, the average peak temperatures of the pellicle composite increased from 507 to 514 and 528 °C. Then, the emissivity of the pellicle composite was calculated using Equation (7). Here, the emissivity of the SiN_x_ thin film was approximately 0.003, and the absorbance in the IR region was close to 0. Therefore, the calculated emissivity of the Ru/SiN_x_ pellicle composite was assumed to be the same as that of the Ru thin film [13]. After the annealing treatment at 300 and 500 °C, the emissivity of the Ru film decreased from 0.48 to 0.46 and 0.43, respectively. From these results, the proportionality between the resistivity and emissivity based on the Lorentz–Drude model was experimentally verified.

## 5. Conclusions

The relationship between the resistivity and emissivity of the Ru thin film according to microstructural changes (grain size) was confirmed. Due to the post-deposition annealing process at 300 and 500 °C, grain growth was observed while the film thickness and surface roughness were unchanged. Reduced grain boundary scattering decreases the resistivity of the Ru thin film. A decreased emissivity of the pellicle was also observed through the heat load test emulating an EUV scanner environment that excludes the cooling effect by heat conduction and convection. From these results, the proportional relationship between the resistivity and emissivity of a thin metal film based on the Lorentz–Drude model was experimentally verified. In this paper, a small-sized pellicle membrane was studied due to the technical difficulties of the pellicle fabrication process, but a similar tendency is expected to be observed in a full-size pellicle which is needed in the EUV lithography process.

As the emissivity data for nanomaterials are currently difficult to obtain, the results of this study provide insight that facilitates the prediction of emissivity from the resistivity, which is relatively easier to measure. Therefore, the emissivity can be improved by changing the microstructure in the direction of increasing thin film resistance.

## Data Availability

The datasets generated and/or analyzed during the current study are available from the corresponding author upon reasonable request.
